# Lenalidomide enhances the efficacy of anti-BCMA CAR-T treatment in relapsed/refractory multiple myeloma: a case report and revies of the literature

**DOI:** 10.1007/s00262-021-02959-8

**Published:** 2021-05-18

**Authors:** Guoxing Zhao, Runhong Wei, Lei Feng, Yi Wu, Feng He, Mingxing Xiao, Zhi Cheng

**Affiliations:** 1 Department of Hematology, Henan Province Hospital of Traditional Chinese Medicine (The Second Affiliated Hospital of Henan University of Traditional Chinese Medicine), Institute of Hematology, Henan University of Traditional Chinese Medicine, Zhengzhou, 450002 China; 2 HRAIN Biotechnology Co., Ltd., Shanghai, China

**Keywords:** Multiple myeloma, Lenalidomide, Chimeric antigen receptor *T* cell, B cell maturation antigen

## Abstract

We report successful clinical experience using anti-BCMA CAR-T combined with lenalidomide in a patient who was refractory to a previous CAR-T treatment. The patient was a 51-year-old man, and was diagnosed with IgD-λ multiple myeloma(MM) in October 2015. 10 courses of chemotherapy including immunomodulators and proteasome inhibitors were used for remission and autologous hematopoietic stem cell transplantation was performed. MM relapsed after 12 months of remission. His disease continued to progress after multiple chemotherapy regimens, mouse anti-BCMA CAR-T and human-derived anti-BCMA CAR-T therapy. After a conditioning chemotherapy regimen of fludarabine and cyclophosphamide, patient took lenalidomide on day -1 and human-derived anti-BCMA CAR-T cells were infused on the next day. He suffered grade 2 cytokine-releasing syndrome(CRS) and grade 3 myelosuppression after infusion, and were resolved after symptomatic treatment. Very good partial response (VGPR) was achieved 14 days after CAR-T treatment, and had been maintained for more than 8 months. We demonstrated for the first time in patients that anti-BCMA CAR-T cell therapy combined with lenalidomide is feasible and effective in the treatment of RRMM. It provides a new strategy for RRMM patients who do not respond to anti-BCMA CAR-T cell therapy alone, and the adverse event is reversible.

## Background

Recent studies have shown that using anti-B-cell maturation antigen chimeric antigen receptor *T* cell (BCMA CAR-T) to treat relapsed/refractory multiple myeloma (RRMM) had achieved impressive results. Studies have reported an overall response rate (ORR) of 64% to 93%, and some patients achieved MRD negative or no abnormality in molecular biology, which indicated deep remissions [1–4]. However, for patients who had no response to anti-BCMA CAR-T therapy alone, the median overall survivals were only 5–9 months [5]. Therefore, new anti-BCMA CAR-T treatment strategies such as dual-target CAR-T therapy, dual-phenotype CAR-T therapy, and combination therapies still need to be explored [3]. In recent years, some animal studies and *in vitro* studies have reported that lenalidomide could enhance the expansion and function of CAR-T cells. However, report of using anti-BCMA CAR-T therapy combined with lenalidomide to successfully treat RRMM in clinical settings has not yet seen [6, 7]. In this article, one case with RRMM treated with anti-BCMA CAR-T cell therapy combined with lenalidomide is presented.

## Case presentation

A 51-year-old male patient visit the hospital in October 2015 with onset of pain in his left lower limb. MRI showed there were multiple abnormal signals in his upper femur on both sides. 76.4% immature plasma cells were detected by morphological examination of the patient’s bone marrow cells and the immunophenotyping by flow cytometry exhibited the expression pattern CD38 +  + , CD138 + , CD19-, CD56-, CD20 + , CD81 + , CD117. His serum M-protein level was 0.75 g/L, and his serum light chain lambda level was 28.7 g/L, the patient was definitely diagnosed with IgD-λ MM. He started treatment with 6 courses of VAD (vincristine, doxorubicin and dexamethasone) and 4 courses of BD (bortezomib, dexamethasone). After reaching VGPR, autologous hematopoietic stem cell transplantation and consolidation chemotherapy were performed on June 17, 2017. In May 2018, the patient’s bone marrow was re-examined. 3.2% plasma cells in nucleated cells of bone marrow and abnormal phenotypes were detected using flow cytometry, which suggested disease recurrence. Despite using BPD (bortezomib, pirarubicin, dexamethasone), BD, PCTD (bortezomib, cyclophosphamide, thalidomide and dexamethasone) and other regimens to treat the relapse disease, the patient’s disease continued to progress. A mouse anti-BCMA CAR-T were treated on December 24, 2018 in a other hospital, but no efficacy showed. After that, 2 courses of DECP (Dexamethasone, etoposide, ifosfamide and cis-platinum)regimen, 2 courses of isazomide plus dexamethasone, 1 course of isazomide plus lenalidomide and dexamethasone were administered. The disease continued to progress.

**Fig. 1:**
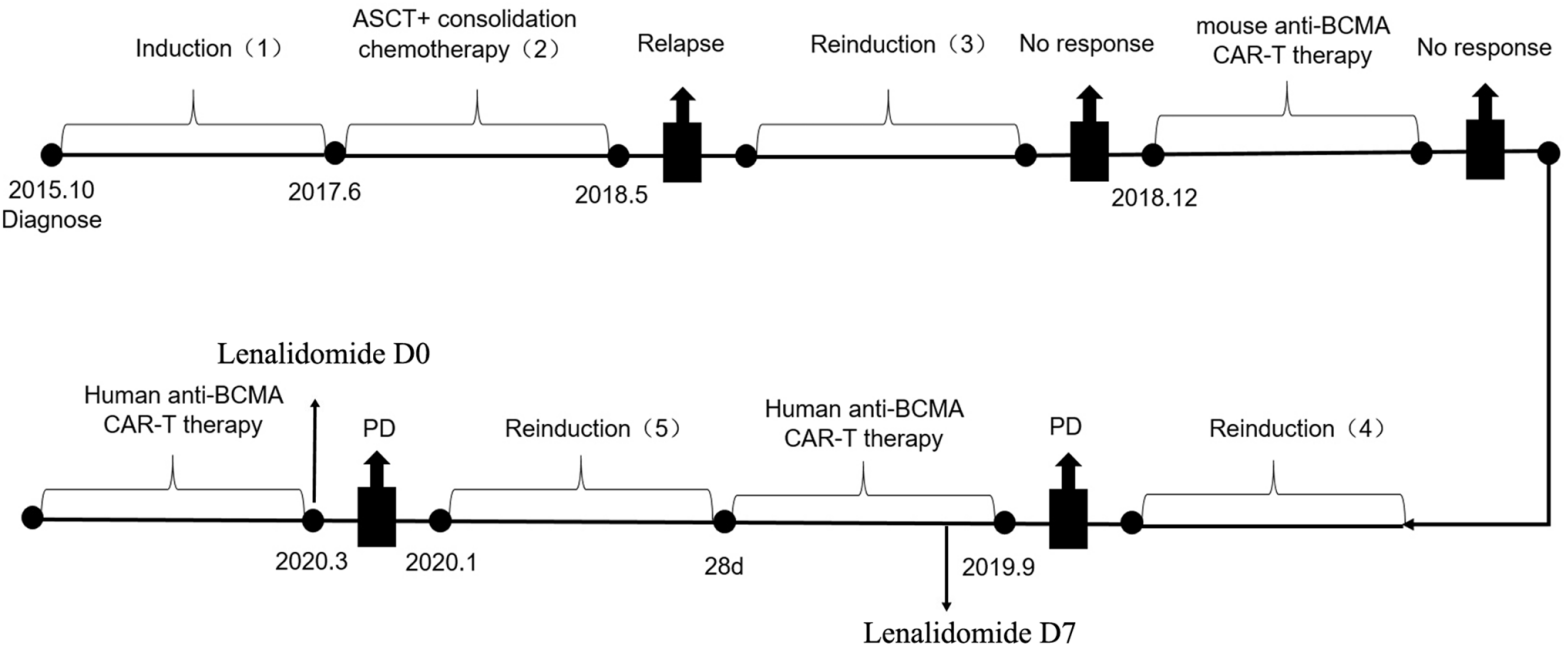
Patient’s diagnosis and treatment timeline. **1** Induction regimen were VAD (vincristine, doxorubicin and dexamethasone) and BD (bortezomib-dexamethasone). **2** Consolidation chemotherapy regimen were Bortezomib and PAD (bortezomib, doxorubicin and dexamethasone). **3** Reinduction regimen were BPD (bortezomib, pirarubicin, dexamethasone), BD, PCTD (bortezomib, cyclophosphamide, thalidomide and dexamethasone). **4** Reinduction regimen were DECP (Dexamethasone, etoposide, ifosfamide and cis-platinum)regimen, isazomide plus dexamethasone, isazomide plus lenalidomide and dexamethasone. **5** Another reinduction regimen were lenalidomide and dexamethasone, ixazomib plus lenalidomide and dexamethasone

**Fig. 2:**
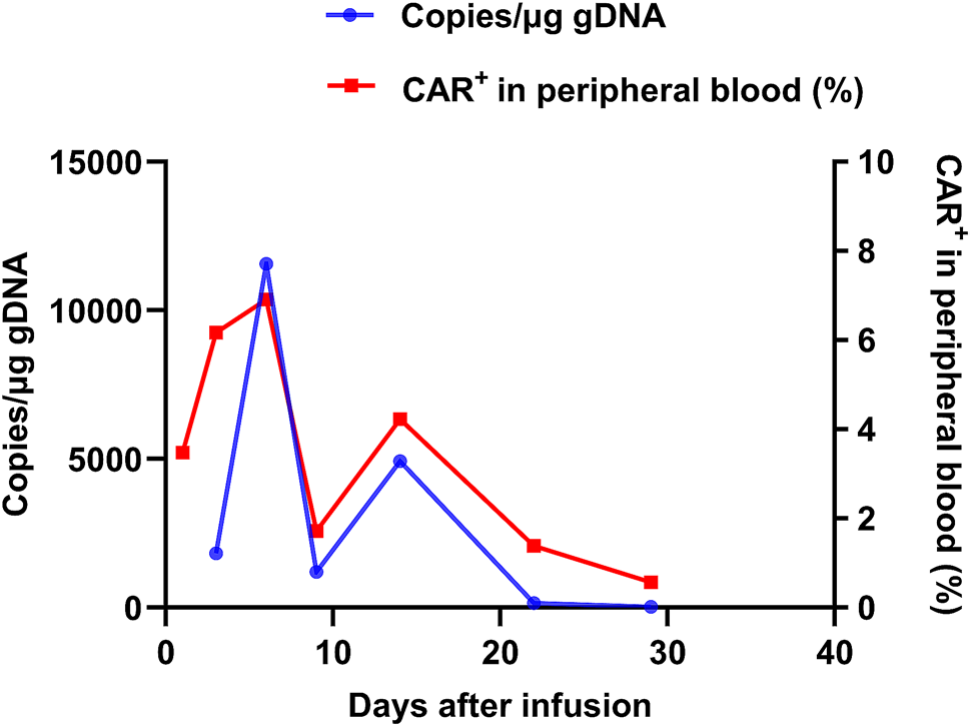
Cell expansion and persistence in peripheral blood after the first infusion of CAR-T cells

In September 2019, bone marrow examination by flow cytometry showed 61.5% plasma cells in nucleated cells of bone marrow, and the proportion of BCMA + plasma cells was 88.36%. the fluorescence in situ hybridization (FISH) of bone marrow indicated CKS1B (1q21) and CDKN2C (1p32.3) genetic abnormalities. The patient was then enrolled in a clinical trial of anti-BCMA CAR-T cell therapy (ClinicalTrials.gov number, NCT04003168) (Fig. 1). He started the lymphodepletion precondition before CAR-T cell therapy on September 25, 2019. Fludarabine 50 mg and cyclophosphamide (CTX) 500 mg were given once a day for total of three days. On September 29, 2019, human-derived anti-BCMA CAR-T cells from Shanghai Hrain Biotechnology Co., Ltd. were infused at a dose of 12 × 10^6^ CAR-T cells/kg. After the infusion of the cells, the patient developed fever with a maximum body temperature of 38.5 °C. After physical cooling, his body temperature returned to normal. The vector copy number of BCMA-CAR reached its peak at day 6, which was 1.16 × 10^4^ copies/μg. The proportion of CAR-T cells in peripheral blood CD3 cells also reached its peak at day 6, which was 6.91% (Fig. 2). On the seventh day after infusion, a peripheral blood smear showed that there were still immature plasma cells, and therefore 12.5 mg of lenalidomide was prescribed orally once a day for 3 weeks. 28 days after CAR-T infusion, the patient’s condition was close to minimal response, with 26.0% of immature plasma cells in bone marrow. He was then given 1 course of lenalidomide and dexamethasoneand 2 courses of ixazomib plus lenalidomide and dexamethasone (Fig. 1).

**Fig. 3:**
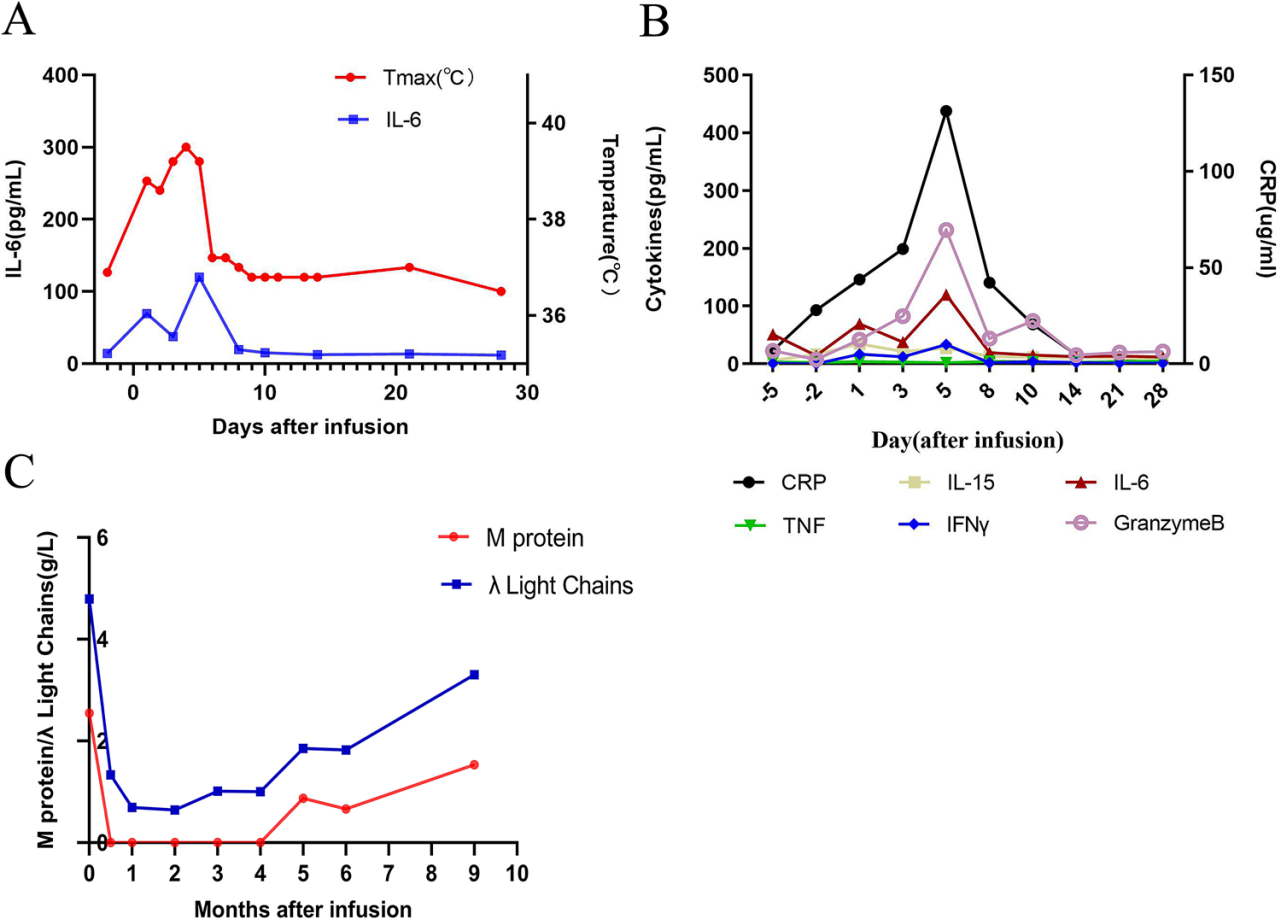
Measures of clinical responses to the second infusion of CAR-T cells. **a** Changes of body temperature after infusion, the plot showed the maximum temperature for each day. **b** Serum cytokines levels and inflammatory markers were measured at the indicated time points after CAR-T infusion. **c** Serum M protein, λ light chains were measured at the indicated time points after CAR-T infusion

**Fig. 4:**
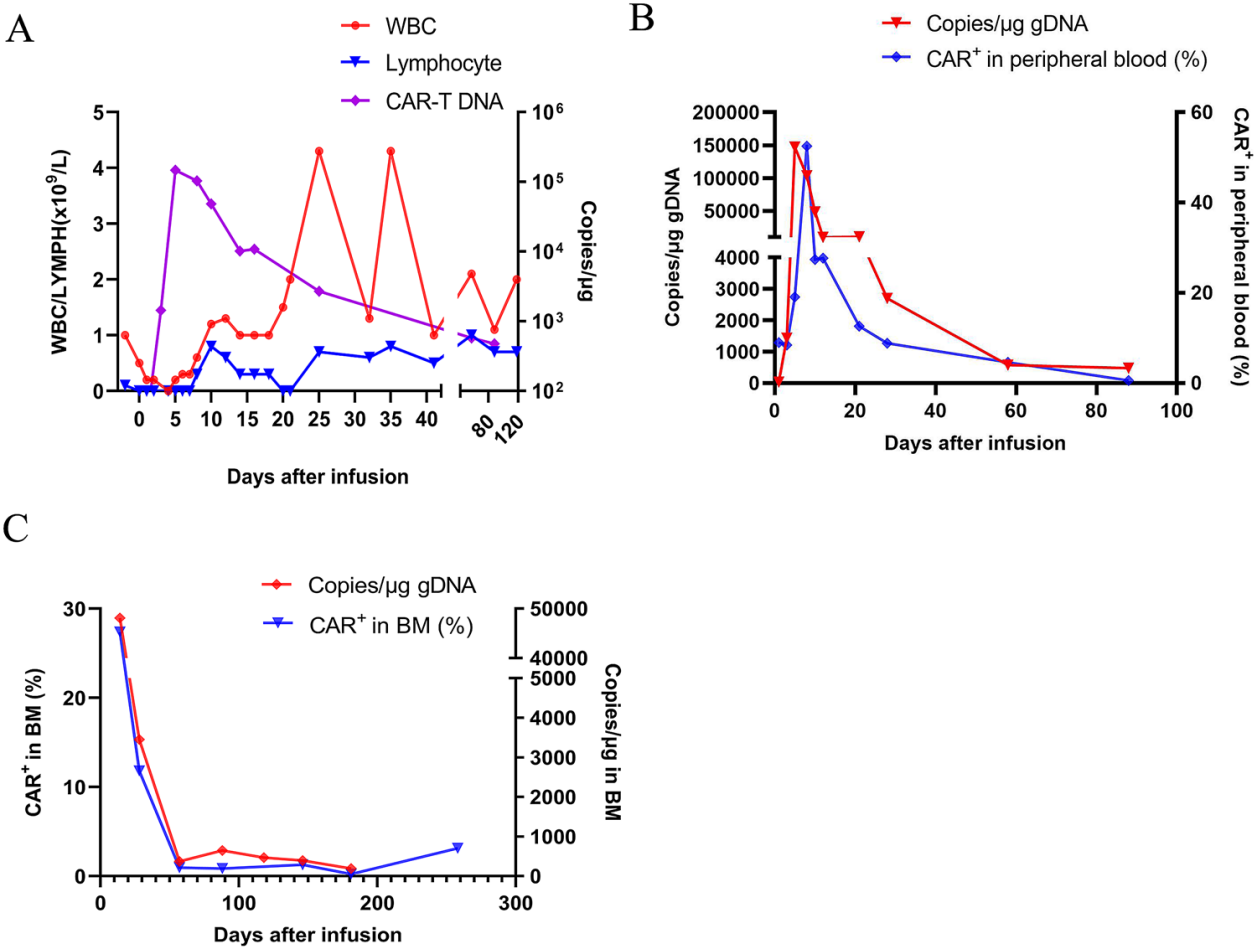
Cell expansion and survival in the body after the second infusion of CAR-T cells. **a** Changes of CAR transgene copies in peripheral blood, and blood cell counts including white blood cell and lymphocyte. **b** Cell expansion and persistence in peripheral blood. **c** Cell expansion and persistence in bone marrow

In January 2020, the patient was admitted to the hospital again due to systemic pain. The bone marrow morphology showed 98% of immature plasma cells and binuclear cells could be observed. The peripheral blood smear showed 18% of immature plasma cells, bone marrow examination by flow cytometry showed 91.2% of plasma cells, and 56.2% of them were BCMA + Plasma cells. Bone marrow FISH indicated CKS1B (1q21) and CDKN2C (1p32.3) genetic abnormalities. PET-CT showed osteolytic lesions in patient’s thoracic spine and right iliac. Under the approval of the hospital ethics committee, lenalidomide combined with anti-BCMA CAR-T therapy was used on the basis of anti-infection, traditional Chinese medicine and supportive treatment. 3 days before CAR-T cells infusion, Fludarabine 40 mg and CTX 400 mg were prescribed for lymphodepletion precondition. On the day right before CAR-T cells infusion, the patient started to take lenalidomide orally at a dose of 25 mg, once a day for 3 weeks. On March 26, 2020, the human-derived anti-BCMA CAR-T prepared by Shanghai Hrain Biotechnology Co., Ltd. was infused at a dose of 10 × 10^6^ CAR-T cells/kg. Recurrent fevers were developed 4 h after the infusion with a maximum temperature of 39.5 °C, the patient was treated according to his symptoms and the fever was relieved on the 7th day (Fig. 3a). According to the ASTCT standard [8], the patient experienced grade 2 cytokine release syndrome (CRS) and no central nervous system toxicity was observed. Grade 3 adverse events that the patient experienced include: neutropenia, low white blood cell count, low lymphocyte count, low platelet count, and anemia. The peak level of IL-6 was detected on the fourth day after infusion to be 119.8 pg/mL (Fig. 3a), and other cytokines such as IFN-γ, Granzyme B, and CRP reached their peaks on the fifth day after infusion (Fig. 3b). The peak copy number of BCMA-CAR in peripheral blood was 1.47 × 10^5^ copies/μg (day 5) (Fig. 4a). Continuous expansion of BCMA CAR-T cells was also observed by flow cytometry, and the highest proportion of BCMA CAR-T cells in peripheral blood *T* cells reached 52.5% (Fig. 4b). The copy number of BCMA-CAR in bone marrow reached 4.81 × 10^4^ copies/μg 14 days after CAR-T infusion, and the proportion of BCMA CAR-T cells was 27.4% as observed by flow cytometry. Both the copy number and the proportion started and continued to decrease after that day (Fig. 4c). Starting from the 4th day after CAR-T cell transfusion, red blood cells and platelets were infused as supportive treatment. 9 days after infusion, since neutrophils (NEC) was lower than 0.2 × 10^9^/L and platelets level was lower than 20 × 10^9^/L, lenalidomide was stopped. Patient’s serum M-protein was detected to be negative 14 days after infusion, and the free lambda light chain level significantly decreased (Fig. 3c). Flow cytometry examination of patient’s bone marrow showed that the proportion of plasma cells was 0.08%, and the efficacy of the treatment was evaluated as MRD-negative VGPR. Two months after CAR-T treatment, the copy number of BCMA-CAR was significantly reduced, and the M-protein and free lambda light chain were kept negative for 5 months (Fig. 3c). At about 9 months after the infusion, the patient's serum M-protein and free lambda light chain levels increased, and the proportion of plasma cells in bone marrow was 4.5%, and 56.1% of them were BCMA + plasma cells. The patient was still on follow-up visits.

## Discussion

The patient we presented was initially diagnosed with IgD-λ RRMM, accompanied by rare complex karyotypes, CKS1B (1q21) and CDKN2C (1p32.3) genetic abnormalities, and high tumor burden. After initial treatment, VGPR was achieved and the patient received autologous hematopoietic stem cell transplantation. 12 months after transplantation, relapse of MM was detected, and the patient was treated with chemotherapy that used proteasome inhibitors such as ixazomib, immunomodulators such as lenalidomide for 12 courses, and the mouse anti-BCMA CAR-T Despite the treatment, the patient's disease continued to progress. He then received the human-derived anti-BCMA CAR -T cell treatment and had only achieved minimal response. The patient’s disease had almost progressed to plasma cell leukemia (18% immature plasma cells in peripheral blood, 98% immature plasma cells in bone marrow), and clinical treatment became very difficult and the following treatment showed no response. We finally decided to use the human-derived anti-BCMA CAR-T treatment combined with lenalidomide, and the patient achieved VGPR which lasted for more than 8 months using this treatment strategy. This case showed that the combination therapy of anti-BCMA CAR-T and lenalidomide had good therapeutic efficacy. Although grade 2 CRS and other adverse events occurred during the treatment, they were reversible.

For RRMM patients who do not respond to anti-BCMA CAR-T treatment, new strategies including dual-target CAR-T, dual-phenotypic BCMA CAR-T, and combination therapies are currently being explored to optimize anti-BCMA CAR-T cell therapies. Among those strategies, successful clinical treatment of RRMM with anti-BCMA CAR-T combined with lenalidomide has not been reported and there is also no clinical data on the treatment of IgD-λ RRMM patients with anti-BCMA CAR-T combined with lenalidomide. Although Mailankody of Memorial Sloan-Kettering Cancer Center (MSKCC) in the United States registered a clinical trial (NCT03070327) which explored using anti-BCMA CAR-T cells together with or without lenalidomide for the treatment of MM in 2017, there had been no data reported in any form so far [6, 7]. In recent years, lenalidomide was proved to enhance the function and expansion of CAR-T in animal studies and *in vitro* studies. Works [9] et al. proved through *in vitro* studies that lenalidomide promoted the secretion of cytokines such as IFN-γ, IL-2 and TNF-α in anti-BCMA CAR-T, and enhanced the function of anti-BCMA CAR-T cells in a dose-dependent manner, which led to increased tumor clearance. Wang [10] et al. found that lenalidomide promoted the expansion of CD8 + CAR-T cells, increased the formation of immune synapses between CAR-T cells and tumor cells, significantly inhibited tumor growth, and extended CAR-T cells’ persistence and survival in tumor-bearing mice (*p* < 0.01). In a study that used anti-BCMA CAR-T and lenalidomide combination treatment to treat MM tumor-bearing mice, lenalidomide was given for 50 days, and it was found that lenalidomide increased the function of anti-BCMA CAR-T in a dose-dependent manner and increased tumor clearance [9]. However, it is worth noting that adding lenalidomide after the peak of anti-BCMA CAR-T cells expansion was reached (14 days after CAR-T infusion) could not improve tumor clearance and survival of tumor-bearing mice. In conclusion, these animal studies and *in vitro* studies have proved that lenalidomide increases the expansion and function of CAR-T, promotes the secretion of cytokines and MM cell lysis, and the timing of adding lenalidomide has an impact on the prognosis of treatment.

As far as we know, this case reported in this article was the first successful clinical treatment case of RRMM patients with anti-BCMA CAR-T combined with lenalidomide. The patient received the first infusion of the human-derived anti-BCMA CAR-T cells at a dose of 12 × 10^6^ CAR-T cells/kg. On the sixth day after the infusion, lenalidomide was added. The therapeutic effect was evaluated 28 days after the infusion, and only minimal response achieved. The possible reason for the low efficacy maybe lower expansion of anti-BCMA CAR-T cells after infusion (the peak of BCMA CAR copy number was 1.16 × 10^4^ copies/μg, which was 35 times to day 1). The second CAR-T cells infusion was accompanied by oral administration of lenalidomide starting from the day before the infusion. The peak copy number of BCMA CAR in peripheral blood on the fifth day after infusion was 1.47 × 10^5^ copies/μg, which was 4494.3 times to day 1. 14 days after infusion, patient had reached MRD-negative VGPR, indicating that lenalidomide enhanced the anti-BCMA CAR-T cells’ expansion and efficacy. As a synergist of anti-BCMA CAR-T, oral tablet formulation of lenalidomide makes its administration very convenient, the dose can be increased or decreased, and the drug can be stopped at any time. However, lenalidomide may need to be applied simultaneously with or in advance of anti-BCMA CAR-T cells in order to have a good synergistic effect, which is consistent with the findings of *in vitro* and animal studies. Certainly, the application of this combination therapy still requires strict clinical trial verification.

Combining anti-BCMA CAR-T cell therapy with lenalidomide improves CAR-T cell therapy’s efficacy of treating RRMM, however this may also increase its toxicity. Compared with the transient fever after the first human anti-BCMA CAR-T infusion, this patient developed grade 2 CRS after the second infusion, symptoms included fever, pancytopenia, and grade 3 bone marrow suppression. Studies indicated that lenalidomide or anti-BCMA CAR-T cell therapy alone can cause pancytopenia and severe bone marrow suppression [11, 12]. The superimposed effect of anti-BCMA CAR-T cell therapy combined with lenalidomide may be a major factor leading to grade 3 bone marrow suppression. Therefore, when using the combination therapy of CAR-T cells and lenalidomide, peripheral blood hemogram should be closely monitored and symptomatic treatment should be actively performed. The molecular mechanism of lenalidomide increasing the expansion and efficacy of anti-BCMA CAR-T cells is not fully understood, but studies have shown that lenalidomide can induce the degradation of the transcription factors Ikaros (IKEF1) and Aiolos (IKEF3) in *T* cells [13]. IKZF1 and IKEF3 in T cells inhibit IL-2 gene transcription, and therefore the degradation of IKEF1 and IKEF3 leads to an increase in IL-2 production, which further leads to the activation of *T* cells and NK cells [14, 15], thereby enhances the expansion and function of anti-BCMA CAR-T cells. However, we evaluated the patient’s IL-2 level 10 days before and after the CAR-T cell infusion, and there was no increase in IL-2 concentration.

In conclusion, even though huge progress has been made regarding RRMM treatment strategies, it is still necessary to explore new therapies for RRMM patients who are resistant to immunosuppressants, proteasome inhibitors, and especially anti-BCMA CAR-T cell therapy. We provided a new treatment strategy which combined BCMA CAR-T cell therapy with lenalidomide for those RRMM patients, and it was showed to be safe and effective through the case presented in this article.

## Abbreviations

BCMA: B cell maturation antigen
CAR: Chimeric antigen receptor
CRS: Cytokine-releasing syndrome
CTX: Cyclophosphamide
MM: Multiple myeloma
MRD: Minimal residual disease
NEC: Neutrophil
ORR: Overall response rate
RRMM: Relapsed/refractory multiple myeloma
VGPR: Very good partial response
